# Salvage Radiosurgery After Brachytherapy for Locally Recurrent Prostate Cancer With Real-Time Adaptive Motion Management Using Previously Implanted Brachytherapy Seeds as Fiducial Markers

**DOI:** 10.7759/cureus.81373

**Published:** 2025-03-28

**Authors:** James B Yu, David J Grew, Charu Singh, Erin Sculley, Aaron Jones, Reshma Munbodh

**Affiliations:** 1 Radiation Oncology, Dartmouth Hitchcock Medical Center, Lebanon, USA; 2 Radiation Oncology, St. Francis Hospital, Hartford, USA

**Keywords:** prostate cancer, robotic radiosurgery, sabr, salvage radiation, sbrt, stereotactic body radiotherapy

## Abstract

Locally recurrent prostate cancer after radiotherapy is a difficult clinical scenario. Focal therapy with stereotactic body radiation therapy (SBRT) is a promising treatment option. In this case report, we present the case of a male patient who underwent low dose rate (LDR) brachytherapy many years ago and experienced a prostate-specific membrane antigen positron emission tomography (PSMA PET) avid local recurrence of prostate cancer with rising prostate-specific antigen (PSA). He was able to undergo salvage CyberKnife (Accuray, Madison, WI) robotic radiosurgery without the need for additional fiducial marker placement by tracking previously implanted brachytherapy seeds. Treatment of 40 Gy in five fractions to the PET avid tumor gross tumor volume (GTV) and 35 Gy in five fractions to the planning target volume (PTV) was successfully delivered with minimal toxicity. The patient tolerated therapy well with excellent urinary and sexual functioning and declining PSA at the last follow-up.

## Introduction

Locally recurrent prostate cancer after radiotherapy is a difficult clinical scenario. There have been multiple prospective series investigating whole gland re-irradiation. These studies have shown high rates of biochemical control, though some patients do experience long-term grade 3 toxicity [1].

More recently, partial gland irradiation with radiosurgery or brachytherapy has been investigated [2]. The French GETUG-AFU 31 demonstrated that partial gland stereotactic body radiotherapy (SBRT) was feasible and safe [3]. At the National Cancer Institute (NCI), a phase I study evaluated five-fraction treatment and found that 40 Gy in five fractions was the maximum tolerated dose [4]. Others have attempted brachytherapy (both high dose rate (HDR) and low dose rate (LDR)), though Grade 3 genitourinary toxicity rates are on the order of 9% [5].

Ideal treatment of prostate cancer is personalized to a patient’s beliefs and desires. Androgen deprivation therapy (ADT) is associated with decision regret, and most men would want an overall survival benefit before choosing ADT [6]. As well, even short-term ADT can cause side effects such as obesity and cardiovascular morbidity, as well as a clear impact on a patient’s quality of life [7].

In this case report, we present the case of a male patient who underwent brachytherapy and experienced a local recurrence of prostate cancer. Prior narrative reviews noted that "fiducial implantation prior to imaging is necessary for CT-based linear accelerator or CyberKnife (Accuray, Madison, WI) treatments" [2]. Here, we report a case where fiducial implantation was not necessary, and the patient was treated successfully with CyberKnife using previously implanted brachytherapy seeds for prostate tracking.

## Case presentation

Some clinical details have been obfuscated for privacy purposes. Our patient was a gentleman in his 60s who presented with a chief complaint of a rising prostate-specific antigen (PSA) approximately 10 years after low dose rate (LDR) brachytherapy for prostate cancer. Originally, he had presented in his 50s with a Gleason 3+4 (Grade group 2) prostate cancer, with pre-treatment PSA <10, and non-palpable (T1c) disease. At the time of initial prostate cancer presentation, staging scans (CT of the abdomen and pelvis and nuclear medicine bone scan) were negative for metastatic disease. 

Initial brachytherapy treatment

He underwent LDR brachytherapy with I-125. A standard brachytherapy technique with ultrasound mapping was performed. Prostate planning treatment volume (PTV) was created from the prostate as outlined on ultrasound with the addition of a 2mm margin in all directions, with the exception of the posterior and superior dimensions. Variseed software was used for treatment planning. The total planned minimum dose to the periphery of the prostate by full decay was 145 Gy. Dose constraints were applied in order to limit the maximum urethral dose to less than 150% of the prescription dose. The maximum dose of the anterior rectal wall was limited to no more than 90% of the prescription dose. 

Pre-loaded I-125 seeds embedded at 1 cm spacing in absorbable suture material were utilized for the implant procedure. A total of 55 I-125 seeds were implanted, each with an activity of 0.42 millicuries per seed, for a total implanted activity of 23.08 millicuries. He tolerated the brachytherapy procedure well.

Post-brachytherapy course and workup upon PSA recurrence

Post brachytherapy, our patient had no long-term genitourinary or gastrointestinal toxicity. The PSA declined to a nadir of 0.1 within two years post treatment. However, in the subsequent seven years (nine years post brachytherapy), PSA steadily rose to 1.1 ng/mL. The PSA rise then accelerated, and in a repeated PSA 12 months after the value of 1.1 ng/mL, his PSA had risen to 5.1 ng/mL.

**Figure 1:**
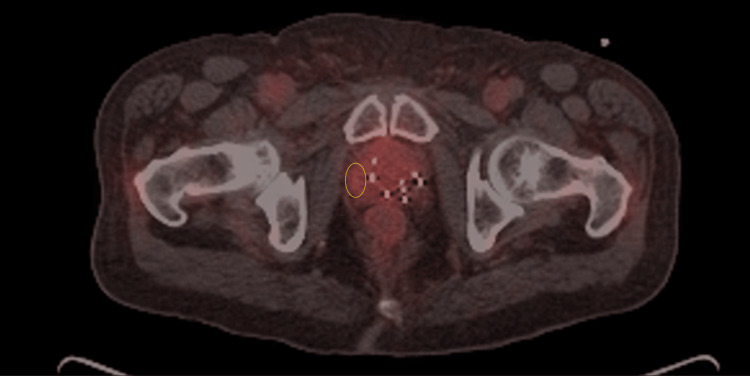
Pylarify PSMA PET showing recurrent disease in right lateral prostate The radiologist-defined PET-avid lesion is circled in yellow PSMA PET: prostate-specific membrane antigen positron emission tomography

At this point, a piflufolastat F18 prostate-specific membrane antigen positron emission tomography (PSMA PET) scan was obtained (Figure 1).

This showed a localized recurrence in the right lateral aspect of the prostate, in an area relatively spared from the presence of brachytherapy seeds. There were no other areas of PET avid disease within the prostate. There was also no evidence of metastatic disease. His urologist recommended he start ADT, but he refused due to concerns about side effects, particularly sexual function. The patient was referred to our radiation oncology practice for a discussion of salvage radiotherapy.

When he presented to our clinic, he was insistent on avoiding ADT as well as any invasive procedures. He was considering no further treatment, as his quality of life was excellent. He was otherwise in excellent health, with no major comorbid illnesses. 

Salvage SBRT treatment planning

We discussed treatment options with him, including whole gland re-treatment as well as partial gland salvage SBRT. Given the potential motion of the prostate [8], we recommended intrafraction motion management. We considered referral for MRI linear accelerator (LINAC)-based radiosurgery but felt it was likely that his previously implanted brachytherapy seeds would obscure the tumor. As well, the patient wished to be treated at a local facility. Other potential motion management solutions available to us (abdominal compression, surface tracking, periodic cone beam CT) were considered inadequate given the precision we needed for partial gland treatment. He also refused to consider a rectal balloon for prostate immobilization.

We therefore decided to attempt treatment using the CyberKnife robotic radiosurgery system, using preexisting brachytherapy markers as fiducial markers. The patient was CT-simulated (feet first, supine) without contrast. The PSMA PET and CT simulation scans were fused, and gross tumor volume (GTV) was outlined to encompass PET avid disease greater than background. Clinical tumor volume (CTV) was expanded from GTV by 1 mm. The PTV was expanded from GTV by 3 mm. The prescription isodose line was 80% of the maximum dose, and the goal of treatment was for at least 97% of the PTV to receive 35 Gy and 100% of the GTV to receive 40 Gy in five treatments, with treatments delivered every other day.

**Figure 2:**
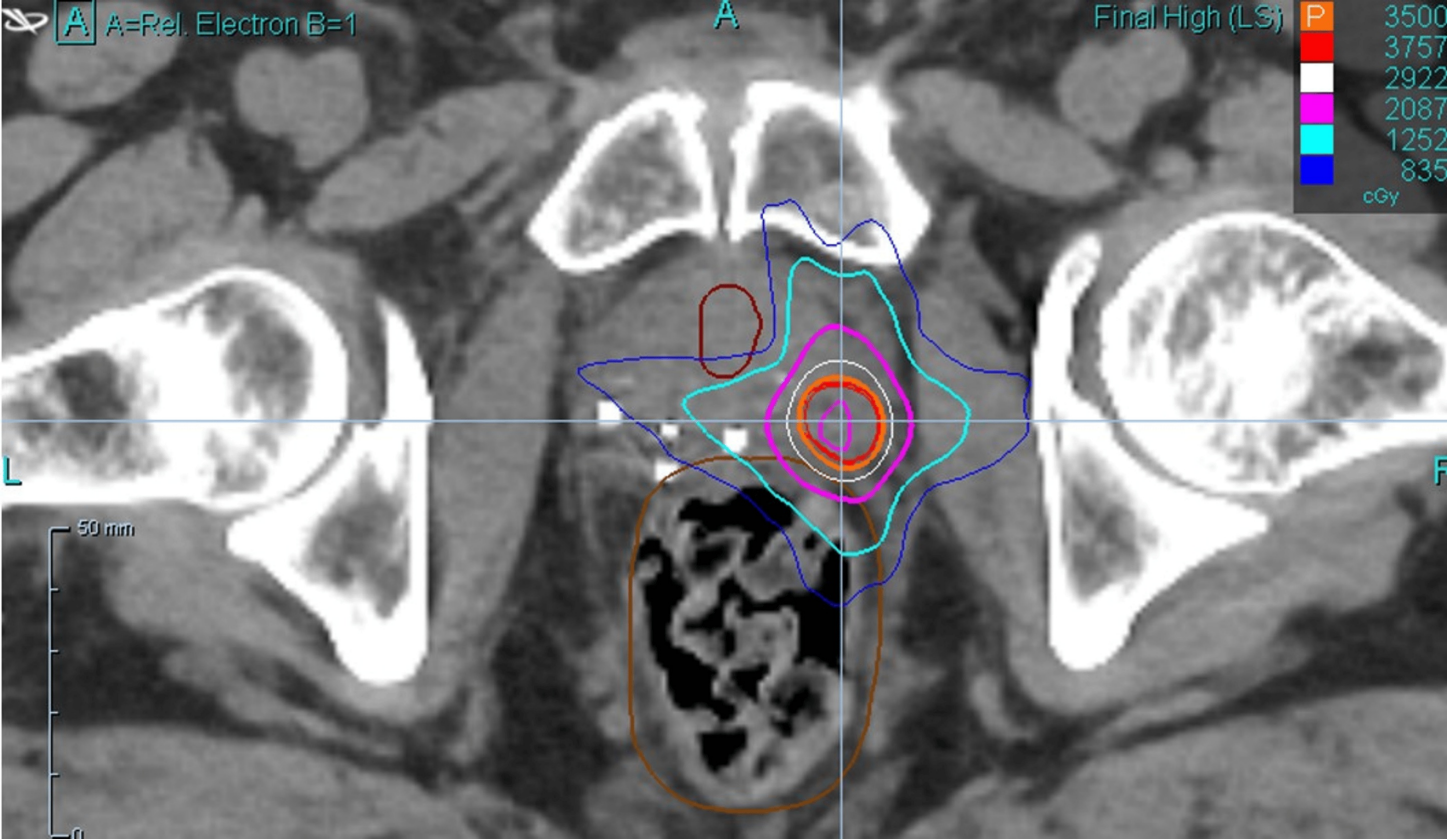
Representative dosimetry (axial view)

**Figure 3:**
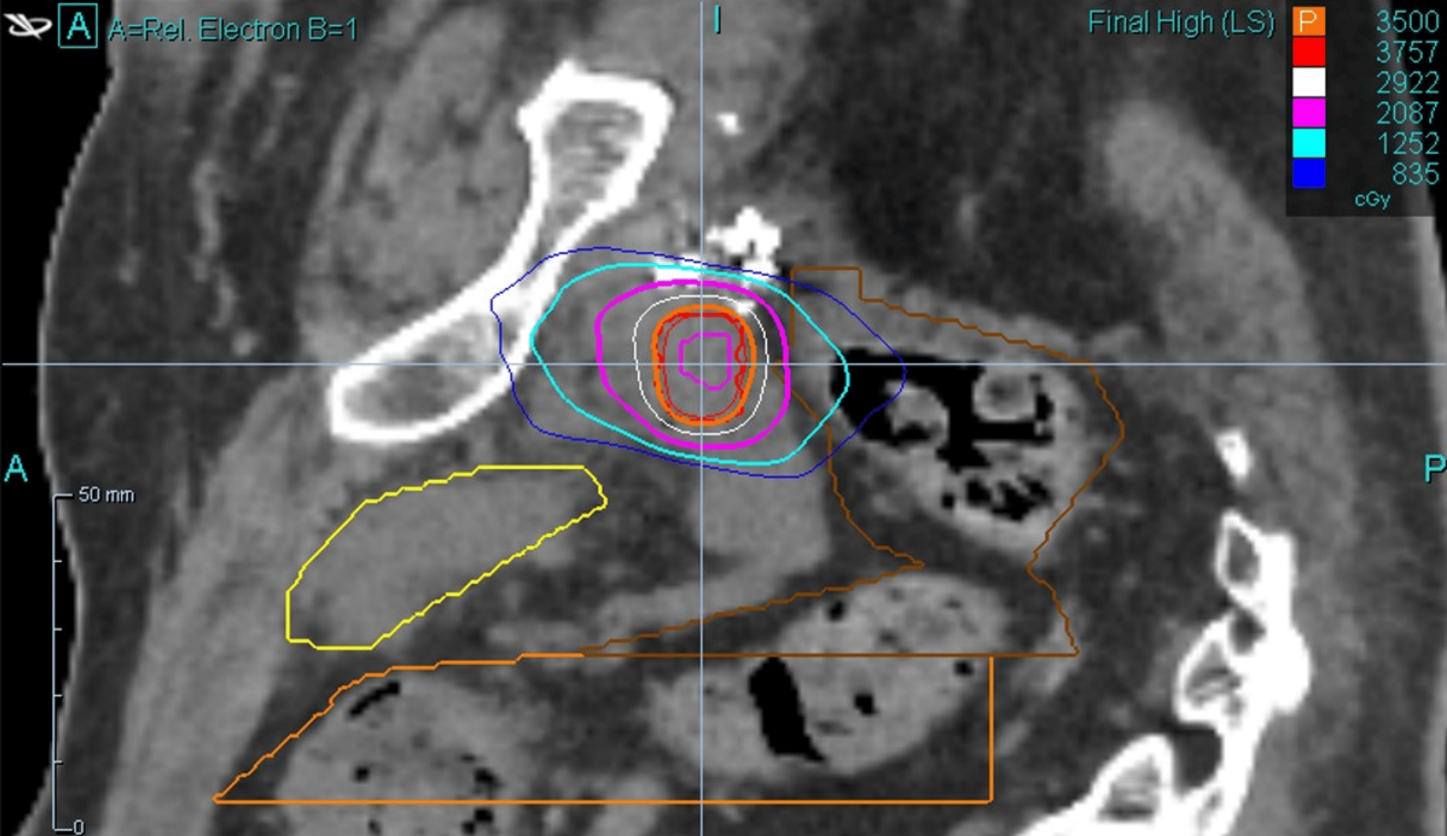
Representative dosimetry (sagittal view)

**Figure 4:**
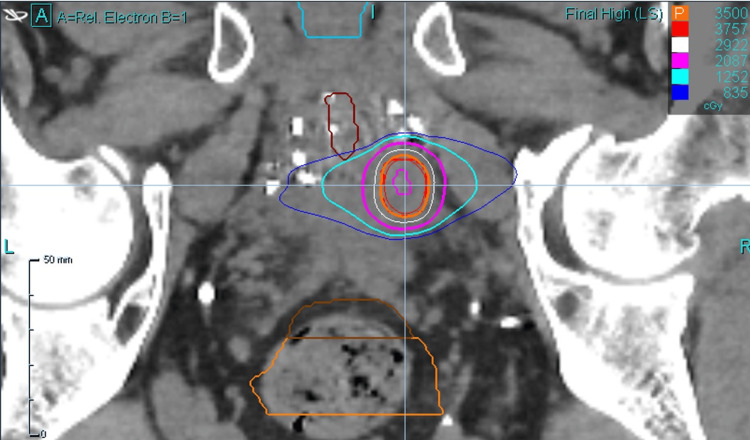
Representative dosimetry (coronal view)

A 29-beam plan was created with 97.12% coverage of the PTV, 100% coverage of CTV with prescription dose, and 100% coverage of the GTV with 40 Gy. The minimum GTV dose was 40.4 Gy. The minimum and maximum PTV doses were 33.6 cGy and 43.7 cGy, respectively. A prostatic urethra avoidance structure was contoured, and it received a maximum dose of 15.0 cGy. An additional planning risk volume (PRV) expansion was not applied to the urethra, but instead, the urethra was contoured generously. The rectum was contoured from the anus to the sigmoid flexure. The maximum rectum dose was 29.1 cGy, with 41% of the rectum receiving 18 Gy. Maximum bladder dose was 21.0 cGy, with 43.4% receiving 19 Gy. Testes were avoided (max dose 0.01 Gy), and the non-rectum bowel received a max dose of 1.2 Gy. Representative dosimetry is shown in Figure 2 (axial view), Figure 3 (sagittal view), and Figure 4 (coronal view).

Robotic SBRT: target tracking using existing seeds

Pre-treatment Phase

**Figure 5:**
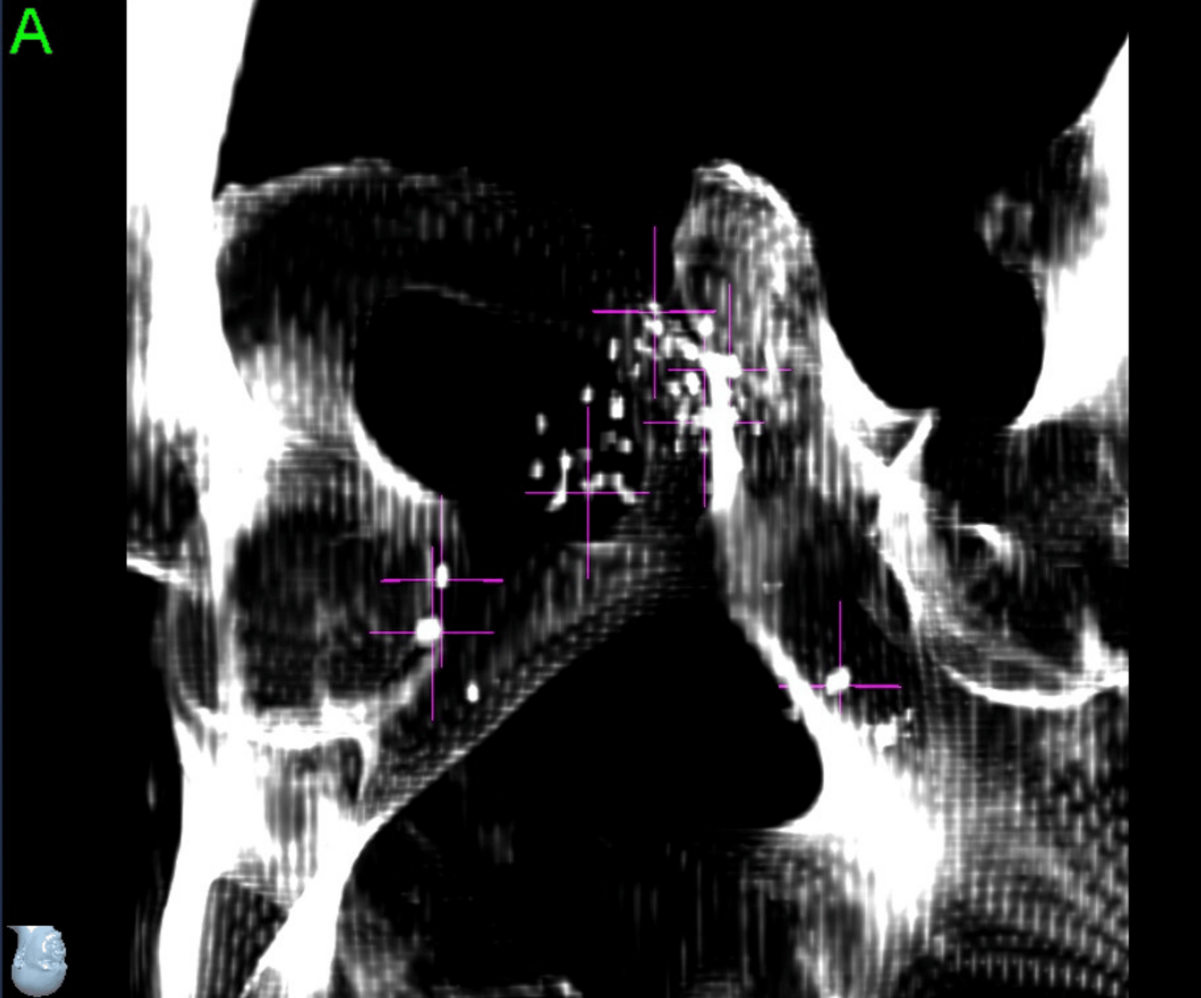
Selecting brachytherapy seeds to use as fiducial markers (left anterior oblique view)

**Figure 6:**
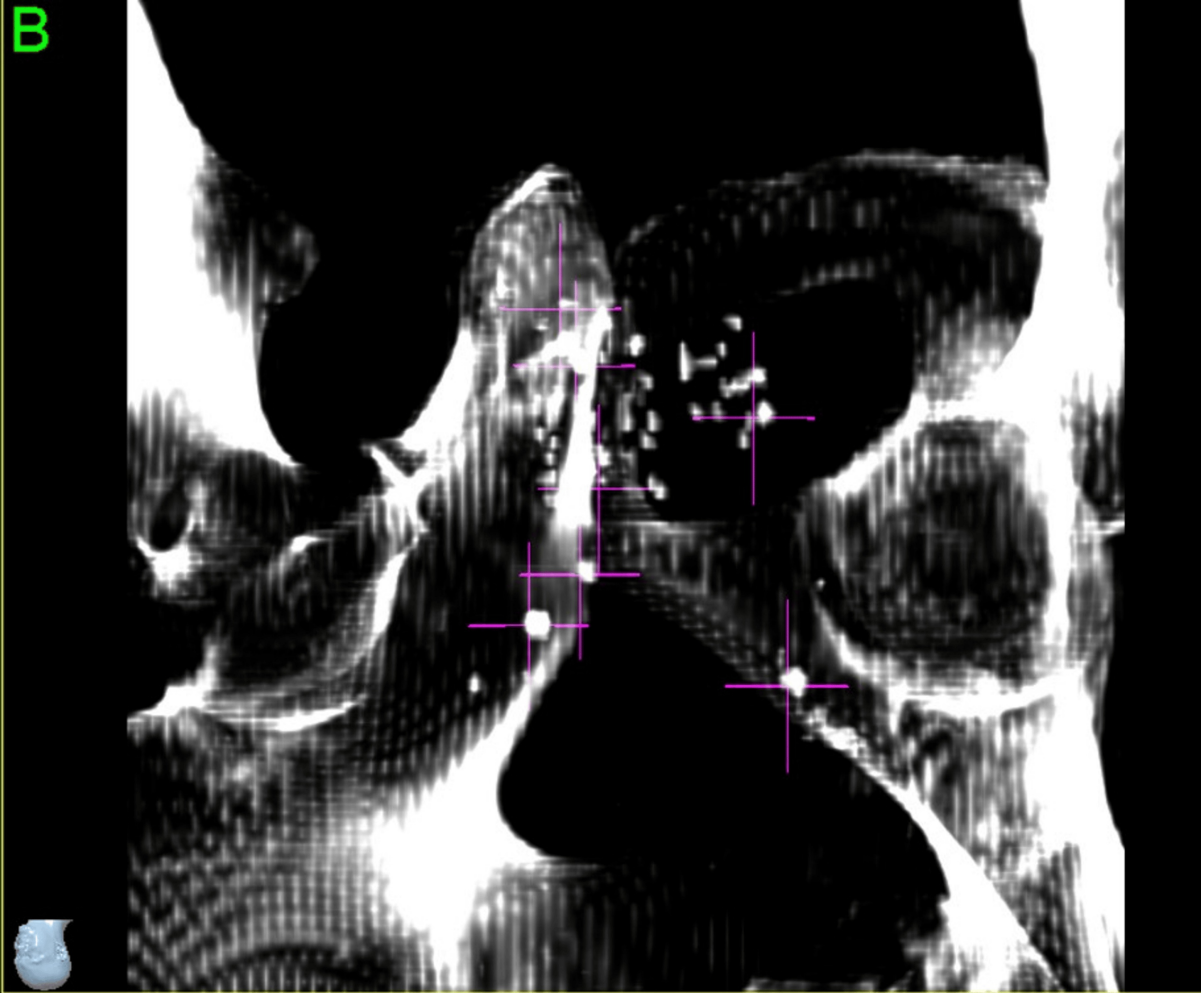
Selection of brachytherapy seeds to use as fiducial markers (right anterior oblique view)

During the planning process, seven total points were defined. Three such points were large calcifications around the periphery of the prostate, which were below the density threshold for the system to track but provided a useful visual aid to confirm the system was locking on to the appropriate seeds. The other four points were what we believed to be the most distinguishable seeds or clusters of seeds: the most superior, the most inferior, the most anterior, and a cluster directly adjacent to the target (Figures 5-6).

Initial Setup Phase 

With the large number of seeds in close proximity to one another at different angles and our previous experience with the system’s tendency to lock on to different points of longer prostate fiducials, we did not want to solely trust the seed points for the larger initial rotational adjustments. Therefore, we made the decision to also have a setup plan for the pelvic bones, which would allow us to apply the initial corrections in all six degrees of freedom and then proceed to our seed tracking points for finer adjustments and periodic intrafraction image tracking at a 30-second interval.

Intrafraction Tracking Phase

Once the patient was well aligned to pelvic bones and calcification aid points, the four identified seed points were evaluated as to which of them were most consistently identified correctly by the software from image to image. At this point, it was determined that only points five and seven were optimal for ongoing tracking; however, point five was the very identifiable “V” cluster directly adjacent to the target, so the entire multidisciplinary team had high confidence that this was a good corollary to the motion of the target. For that reason, the decision was made to proceed with tracking two seed points and monitoring only the translational intrafraction motion. During the course of treatment, while tracking points five and seven, all deviations were less than 0.5 mm.

**Figure 7:**
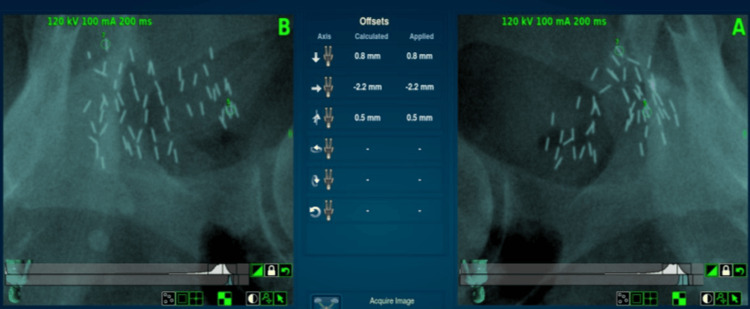
Seed tracking during treatment

The patient completed treatment without delay, and seeds were successfully tracked (Figure 7). 

Post-salvage SBRT Outcome

During the course of treatment, the patient experienced mild dysuria that did not require any medications or treatment modification. 

At one month post treatment, PSA had declined from 5.1 ng/mL pretreatment to 1.2 ng/mL. By four months post treatment, he reported an International Prostate Symptom Score (IPSS) score of 0 (no urinary symptoms) and a Sexual Health Inventory for Men (SHIM) score of 22 (mild erectile dysfunction). He was very pleased with the results of the treatment. The PSA had further declined to 0.7 ng/mL.

## Discussion

Salvage radiosurgery for prostate cancer after prior LDR brachytherapy presents unique challenges for radiation oncology. Low-dose-rate brachytherapy results in high-dose inhomogeneity and normal tissue dose [9]. Instrumentation of the prostate may be difficult given the potential for rectal fistula if the rectum is violated even in biopsy after radiotherapy [10]. Salvage surgery has a high risk for severe gastrointestinal and genitourinary morbidity [11].

Standard therapy for biochemical recurrence after radiotherapy is ADT, which can be life-long. Treatment that is localized, noninvasive, and minimally impacts quality of life is very desirable for patients, given the often-asymptomatic nature of biochemical recurrence. With advances in prostate PET imaging, visualizing the area of recurrence is possible.

Magnetic resonance imaging LINAC-based radiotherapy is a promising technology for the treatment of prostate cancer; however, it is potentially limited when it comes to the treatment of recurrent disease in the setting of brachytherapy due to image artifacts [12].

A platform that can track brachytherapy seeds using continuous motion management is ideal for the treatment of small lesions within the prostate, given breathing motion and setup uncertainty. Without continuous motion management, other options for the treatment of small lesions within the prostate are to immobilize the prostate with a rectal balloon, apply HDR or LDR salvage brachytherapy, or attempt focal ablation of the lesion [5, 13, 14]. For our patient who wished to avoid even minimally invasive treatment, CyberKnife robotic radiosurgery using existing brachytherapy seed tracking offered millimeter precision and excellent initial PSA response with minimal treatment-related toxicity. CyberKnife tracks metal markers (typically fiducials) using orthogonal X-rays. Three fiducials are needed to track translation and rotation, whereas two fiducials can be used if rotation is deemed to be minimal or if the lesion is reasonably bracketed between the markers. As the prostate lesion we were targeting was on the periphery of the prostate, and the targeted lesion was very close to one of the fiducials, we felt that rotation of the prostate on the axis of the theoretical line connecting the fiducials would be minimal. We were gratified by the rapid PSA response and lack of treatment toxicity, which would be consistent with ablative treatment to an accurately localized and tracked lesion.

## Conclusions

In this case report, we describe the successful treatment of a local recurrence of prostate cancer after brachytherapy using robotic radiosurgery. Existing brachytherapy seeds were used for target tracking. The CyberKnife robotic radiosurgery platform is ideally suited for this method of treatment.
